# Effect of Sheet Properties of Cellulosic Polyglycidyl Methacrylate-Grafted Fibers in a Cationic Polyacrylamide/SiO_2_/Anionic Polyacrylamide Retention Aid System

**DOI:** 10.3390/polym16121678

**Published:** 2024-06-13

**Authors:** Yueyue Wang, Pu Ma, Jun Huang, Lifang Guo, Yu Wang, Huamin Zhai, Hao Ren

**Affiliations:** 1 Jiangsu Provincial Key Lab of Sustainable Pulp and Paper Technology and Biomass Materials, Nanjing Forestry University, Nanjing 210037, China; wyyue0328@163.com (Y.W.); yuwang@njfu.edu.cn (Y.W.); hzhai@njfu.edu.cn (H.Z.); 2 Jining Nantian Nongke Chemical Co., Ltd., Jining 372200, China; mapu@nt-chemical.com (P.M.); huangjun@nt-chemical.com (J.H.); guolf@nt-chemical.com (L.G.)

**Keywords:** cellulose fiber, GMA, CPGMAs, papermaking, paper properties, CPAM/SiO_2_/APAM

## Abstract

As increasing fiber hydrophobicity can significantly improve the paper dewatering process, we found that replacing SBKP and HBKP with 0.5% superhydrophobic CPGMA can significantly improve the dewatering of paper sheets. Therefore, it can be concluded that if CPGMA has little effect on paper properties, it will have potential industrial value in the papermaking industry. Consequently, it is necessary to further study the effect of the CPGMAs@CPAM/SiO_2_/APAM system on paper properties. To evaluate the application potential of the system in the papermaking industry, we investigated the effects of CPGMAs, which replaced the fibers in the stocks, on the paper properties in the CPAM/SiO_2_/APAM system. The findings demonstrate that as the CPGMA replacement increased, the paper’s tensile strength, bursting strength, tear resistance, and folding endurance all declined. The trend can be segmented into two phases: a rapid decrease for substitution amounts below 0.5% and a gradual decline for substitution amounts exceeding 0.5%. When replaced with a small amount of CPGMAs, there was a negligible effect on these properties. Second, the paper air permeability increased with the CPGMA substitution amount in the stock. Furthermore, the trend of paper air permeability can be divided into two stages—a rapid stage with a substitution amount of <0.5% and a slow stage with a substitution amount of >0.5%. A small amount of CPGMAs could distinctly improve the paper’s air permeability. Third, CPGMAs, which replaced fibers in the stock, minutely affected the paper formation. A small amount of CPGMAs substantially boosted the efficacy of the process of paper manufacture and certain characteristics of the paper, and it had a negligible impact on the strength of paper. The CPGMAs@CPAM/SiO_2_/APAM technology has the potential to improve the retention and filtration performance of CPAM/SiO_2_/APAM.

## 1. Introduction

As a cornerstone of traditional manufacturing sectors, the papermaking industry holds a significant role in the global economy. Paper industry consumption has gradually shifted to the Asia–Pacific region. In 2009–2018, China’s paper demand growth was higher than that of other countries in the world, with a net growth of 2 million tons; in addition, according to statistics, China’s pulp and paper products industry in 2023 had a pulp, paper, and other output of 291.39 million tons, an increase of 2.63%. The dewatering ability is a significant property in the papermaking process. The dewaterability in papermaking is affected by factors such as the nature of the fiber furnishings, papermaking equipment, and wet-end chemical additives. The addition of wet-end chemical additives generally improves the dewatering efficiency in the papermaking process. The large-scale and high-speed development of paper machines requires wet-end retention and drainage systems [1,2,3,4]. New wet-end chemical programs are based on the interaction between two components, typically a high-mass cationic polymer and a negatively charged mineral “microparticle” [5,6,7]. The microparticle retention and filtration aid system is a highly prevalent approach [8,9,10,11]. A common microparticle retention and filtration aid system for industrial applications is the cationic polyacrylamide (CPAM)/colloidal silica (SiO_2_)/anionic polyacrylamide (APAM) system, which possesses the ability to boost both dewatering and fine-particle retention, and it is represented as CPAM/SiO_2_/APAM [12]. Enhancements in the retention and filtration performance of CPAM/SiO_2_/APAM can promote technological progress in papermaking.

Various technologies, including AKD and rosin, are utilized to achieve a hydrophobic effect. From the perspective of green papermaking and the long term, polymer grafting technology has great advantages. Considering that an increase in the fiber hydrophobicity may enhance the dewatering ability during the process of creating paper, we attempt to enhance the hydrophobic properties of the fiber in order to boost the dehydration efficiency of the CPAM/SiO_2_/APAM system. We established a method for synthesizing cellulosic polyglycidyl methacrylate-grafted fibers (CPGMAs) with glycidyl methacrylate (GMA) as a monomer and Fe^2+^-TDO-H_2_O_2_ as an initiator system [13]. The suggested technique exhibits significant monomer conversion, grafting efficiency, and grafting percentage. The CPGMAs synthesized using this method possess strong hydrophobicity and stiffness [14]. We further investigated the effects of the substitution of CPGMAs in the stock on the dewatering and retention aid performance of papermaking using different proportions of CPGMAs to substitute the corresponding SBKP and HBKP of the stock in the CPAM/SiO_2_/APAM system. We discovered that the performance of the stock increases with the increase in the substitution amount of CPGMAs. Notably, only 0.5% of CPGMAs can further improve the dewatering capacity of the CPAM/SiO_2_/APAM system [15]. We speculate that the CPAM/SiO_2_/APAM system containing a small amount of CPGMAs (CPGMAs@CPAM/SiO_2_/APAM) possesses the potential for industrial application provided it has a limited impact on the sheet properties. Therefore, it is necessary to further study the effects of the CPGMAs@CPAM/SiO_2_/APAM system on sheet properties.

The formation and physical strength are the most basic properties of sheets, closely related to the characteristics of the fibers themselves. In this study, we used polyglycidyl methacrylate-grafted softwood kraft pulp fibers (S-CPGMAs) and polyglycidyl methacrylate-grafted hardwood kraft pulp fibers (H-CPGMAs) with different substitution amounts, replacing SBKP and HBKP, respectively, in the mixed-pulp paper stock (MP) in the CPAM/SiO_2_/APAM system. This study is a continuation of previous research [15] aimed at enabling industrial applications. The objective is to reveal how the different substitution amounts of CPGMAs influence the quality of sheet formation, paper physical strength, and air permeability, to determine the potential of the CPGMAs@CPAM/SiO_2_/APAM system in industrial applications, and to promote the application of the CPGMA@CPAM/SiO_2_/APAM system in papermaking.

## 2. Materials and Methods

### 2.1. Materials

Commercially accessible varieties of pulp boards were hardwood bleached kraft pulp (HBKP, Storaenso, Shanghai, China), softwood bleached kraft pulp (SBKP, Arauco, Santiago, Chile), and bleached chemical–thermal–mechanical pulp (BCTMP, Gold Leopard, Jingui Pulp and Paper Co., Ltd., Guangxi, China); ammonium ferrous kraft ((NH_4_)_2_FeSO_4_-6H_2_O, FAS, 99.5%) and thiourea dioxide (CH_4_N_2_O_2_S, TDO, 98.0%), both A.R. grade, were acquired from Aladdin Biochemical Technologies Co. in Shanghai, China; glycidyl methacrylate (GMA, 97% with stabilizer 100 ppm MEHQ) was acquired from Maclean Biochemical Technologies Co. in Shanghai, China; hydrogen peroxide (H_2_O_2_, 30%, A.R. grade) was acquired from Wokai Biotechnology Company in Shanghai, China; and ground calcium carbonate (GCC, Hydrocarb 75F, Omya, Shanghai, China), cationic starch (CS, Chargemaster L340, Grain Processing Corp, Muscatine, IA, USA), cationic polyacrylamide (CPAM, Percol 292, Ciba Specialty Chemicals Ltd., Basel, Switzerland), colloidal SiO_2_ (SiO_2_, Fennosil 2180, Kemira Oyj, Helsinki, Finland), and anionic polyacrylamide (APAM, EFO 2109-A, SNF, Andrézieux, France) were industrial supplies.

### 2.2. Synthesis of CPGMAs

**Figure 1:**
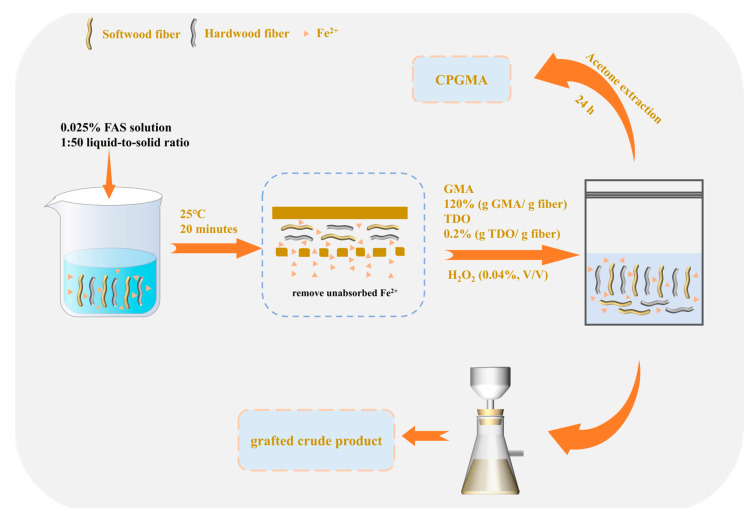
Schematic diagram of CPGMA synthesis method.

CPGMA preparation and analysis were performed according to our previous methods [13,14,16]. As shown in the following Figure 1, after the pulp fibers were measured, they were mixed with a recently made 0.025% FAS solution at a 1:50 liquid-to-solid ratio. The mixture was well mixed and allowed to macerate at 25 °C for 20 min. The fiber samples were properly cleaned to get rid of any unabsorbed Fe^2+^ after the reaction was complete. After moving the fiber-containing Fe^2+^ into a container, the pulp concentration was adjusted to 6% by adding the necessary amount of distilled water. After complete mixing, GMA at 120% (g GMA/g fiber) and TDO at 0.2% (g TDO/g fiber) were added in that order, followed by H_2_O_2_ (0.04%, *v*/*v*). The mixture was thoroughly mixed, and the temperature was increased to 65 °C for a duration of five minutes. At the end of the reaction, the grafted crude product of the grafting process was meticulously washed with deionized water. Acetone was utilized for a whole day to remove the crude product of the grafting process. CPGMA was the copolymer that was left over after the homopolymer was eliminated. The kraft pulp graft fibers that were bleached for hardwood and softwood purposes are referred to as H-CPGMAs and S-CPGMAs, respectively.

The following formulas were utilized to determine the monomer conversion (*C*) and grafting efficiency (*E*):

$$E\left(\%\right)=\frac{m_{2}-m_{0}}{m_{1}-m_{0}}\times 100$$

$$C\left(\%\right)=\frac{m_{1}-m_{0}}{m_{m}}\times 100$$

where m_0_ represents the initial fiber mass; m_1_ denotes the total polymer mass, encompassing homopolymers and copolymers; m_2_ signifies the copolymer mass post acetone extraction to eliminate homopolymers; and m_m_ stands for the original GMA monomer mass.

### 2.3. Stock Preparation

Handsheet stock preparation: A mixed pulp consisting of 28% SBKP, 52% HBKP, and an additional 20% BCTMP, also called MP, was used to investigate the impact of grafted fibers on the characteristics of sheets and the papermaking process. The SBKP, HBKP, and BCTMP pulps had respective freeness values of 380 mL, 375 mL, and 300 mL. The freeness of S-CPGMA and H-CPGMA at 690 mL is identical. SBKP and HBKP were replaced in the mixed pulp in proportions of 0.5%, 2.0%, 5.0%, and 10.0% to look into how the efficiency of the papermaking process is affected by the replacement of graft fibers. The drainage and retention assistance system used in this study is called CPAM/SiO_2_/APAM. Samples devoid of both CPAM/SiO_2_/APAM and GCC are referred to as MP, samples containing GCC alone are referred to as MP + GCC, and samples including both CPAM/SiO_2_/APAM and GCC are referred to as MP + GCC + CPAM/SiO_2_/APAM. 

### 2.4. Handsheet Formation

The process of creating the sheet involved first adding twenty percent GCC (g GCC/g MP) and one percent CS (g CS/g MP) to the paper stock pulp one at a time, separated by three minutes. Subsequently, an additional 30 s was allocated to the blend, incorporating 300 ppm of CPAM, 3600 ppm of SiO_2_, and 250 ppm of APAM. There was 0.20% pulp consistency. The suspension was then filtered on a Kaiser Rapid Sheet Former (RK-2A, Austria) and dried for seven minutes at 96 °C in a vacuum. Basis weight was 60 g/m^2^. Handsheet stock compositions are indicated in Table 1. 

### 2.5. Handsheet Formation Measurement

After a constant temperature (23.0 ± 1.0 °C) and humidity (50.0 ± 2.0%), the handsheet was put into a paper formation meter (LAD07, Taizhou, China) for the formation measurement. The formation of each sheet was measured in 5 different areas on the front and back of 5 handsheets, and the average value was computed. 

### 2.6. Water Contact Angle

Utilizing the static water contact angle, the hydrophobicity of the pulps and CPGMAs was evaluated. A contact angle meter (CAM200, KSV Instruments Ltd., Gothenburg, Sweden) was used to measure the water contact angles (WCAs) of SBKP, HBKP, and CPGMAs using 5 μL deionized water droplets at room temperature. The average of at least five droplets at various places throughout the samples is what determines the value of the WCAs [4].

### 2.7. Handsheet Physical Strength

The tensile index, breaking index, tearing index, and folding resistance of the handsheet were determined according to TAPPI T 404, TAPPI T 403, TAPPI T 494, TAPPI T 414, and TAPPI T 511, respectively.

### 2.8. Handsheet Air Permeability

The air permeability of the paper was measured according to the TAPPI T 536 standard [17]. Burr breathability meter (JH-TQD-X, Shandong Annimet Instrument Co., Ltd., Qingdao, China) was used for measurement at room temperature with a vacuum degree of 1kPa, sample area of 10 cm^2^, and test time of 60 s. Testing was carried out 5 times in different positions of each paper sheet, and the average value was taken.

### 2.9. Data Analysis

Origin 2021 software data analysis and graph processing tools were used to perform data analysis.

## 3. Results and Discussion

### 3.1. Properties of CPGMAs

The synthesized S-CPGMAs and H-CPGMAs showed high values for grafting percentages, grafting efficiencies, and monomer conversions, with values of 120% and 121%, 96% and 97%, and 94% and 94%, respectively. This is mainly due to the strong adhesion properties of the cellulose surface, which is because the hemicellulose inside the fiber can form hydrogen bonds with the hydrophilic cellulose surface, which is conducive to interaction [18]. Furthermore, when grafting to other materials, the adhesive strength of the grafted polymer/cellulose interface is very advantageous [19]. The water contact angles (WCAs) of both the SBKP and HBKP pulps were 0°, indicating extreme hydrophilicity. The WCAs of both the S-CPGMAs and H-CPGMAs were 111.3° and 79.3°, respectively, showing distinct improvement in the hydrophobicity of SBKP and HBKP after the grafting. These results are consistent with our previous research [14]. The distinct improvement in the hydrophobicity undoubtedly benefits the water filtration performance [15] and weakens the bonding performance between fibers.

### 3.2. Impact of CPGMA Substitution on Sheet Formation

**Figure 2:**
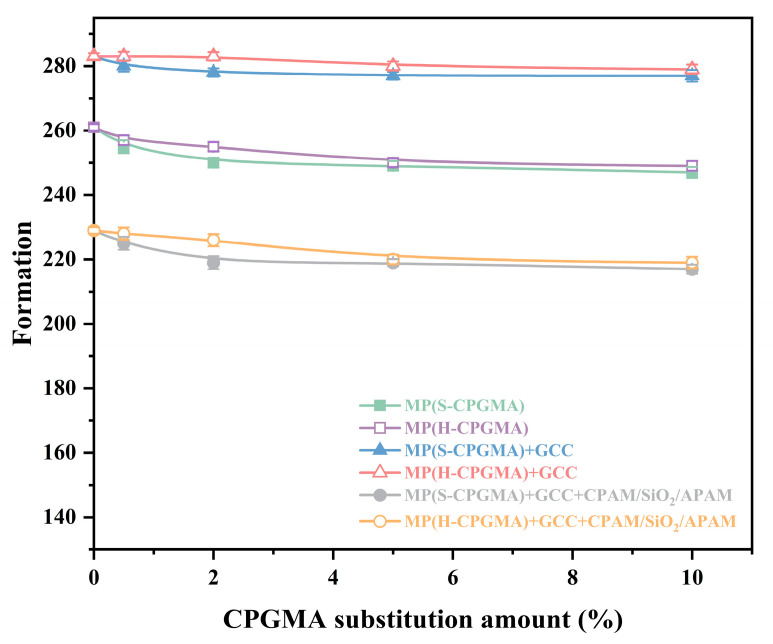
Effect of CPGMA substitution on the sheet formation. (The abbreviations are defined in Table 1 of Section 2.4).

Paper formation has an impact on the overall characteristics of the paper in addition to its appearance. The formation is closely related to the effects of fluid dynamics, wave filtering, flocculation, shearing, and turbulence. The effects of CPGMA substitution on paper formation are depicted in Figure 2. The paper formation is affected by the filler retention and fiber flocculation. As seen from Figure 2, without using CPGMAs, the handsheet formed by MP + GCC possesses the best value, followed by the handsheet formed by MP and that formed by MP + GCC + CPAM/SiO_2_/APAM, which exhibits the worst value. The use of CPGMAs is considered for the comparison of six types of stock, demonstrating the same outcome as mentioned above. This may be primarily attributed to the small GCC size in the handsheet, which evenly distributes and fills in the small gaps between the fibers under the action of fluid dynamics to improve the handsheet formation. However, the CPAM/SiO_2_/APAM system causes a partial flocculation of paper stock fibers, resulting in an uneven fiber distribution and reducing the formation of handsheets.

From Figure 2, it can be found that although there is a tendency for the formation to decrease as the CPGMA substitution amount increases, the effect is negligible, especially when the substitution amount is low. Even when the CPGMA substitution amount is as high as 10%, the formation index of the handsheets formed by the six stocks only decreases by 5.4%, 4.6%, 2.1%, 1.4%, 5.2%, and 4.4%. Moreover, the effect of H-CPMGAs on the decreased formation is slightly smaller than that of S-CPGMAs, which may be primarily attributed to the fact that S-CPGMAs are longer and are prone to entanglement and flocculation. In conclusion, the substitution of fibers in the stock with CPGMAs has a minimal impact on the sheet formation.

### 3.3. Effects of CPGMA Substitution on Sheet Physical Strength

#### 3.3.1. Tensile Strength

**Figure 3:**
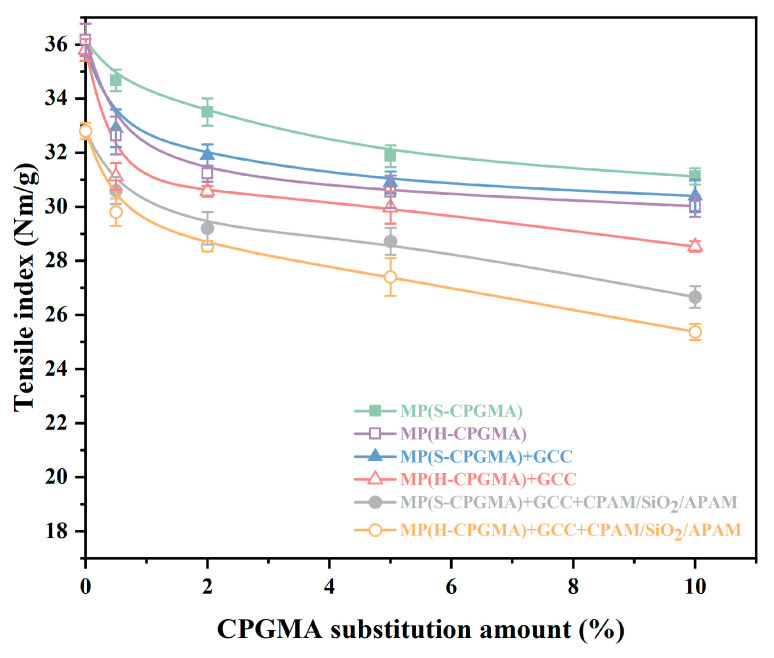
Effect of CPGMA substitution on the sheet tensile strength. (The abbreviations are defined in Table 1 of Section 2.4).

The bonding force between fibers, fiber length, fiber interweaving arrangement, and fiber strength are the primary determinants of the tensile strength of paper sheets. Figure 3 shows the effects of CPGMA substitution on the handsheet tensile strength. When the CPGMA substitution amount is zero, the tensile strength of the handsheet formed by MP is the highest, followed by that of the handsheet formed by MP + GCC. Meanwhile, the tensile strength of the handsheet formed by MP + GCC + CPAM/SiO_2_/APAM is the lowest. In general, the handsheet tensile strength decreases with GCC addition and the application of the CPAM/SiO_2_/APAM system. The GCC between the handsheet fibers reduces the hydrogen bond number, leading to a decrease in the handsheet strength. The retention of GCC is the highest in the CPAM/SiO_2_/APAM system, leading to a more significant decrease in the hydrogen bond number between the fibers.

From the comparison of six stock systems, it was found that the CPGMA substitution amount ranges from 0% to 10%, and the sheet tensile strength decreases as the CPGMA substitution amount increases. There are two phases to the transition process that are characterized by a sharp fall prior to the substitution amount of around 0.5% and a slow decline after the substitution amount of approximately 0.5%. This trend may be attributed to the fact that when the substitution amount is low (<0.5%), the hydrophobic fiber CPGMAs possess a good dispersion and are well distributed among the stock fibers. This might successfully prevent hydrogen bonds from forming between the stock fibers and thus significantly weaken the sheet tensile strength. However, when the substitution amount is high (>0.5%), the hydrophobic fiber CPGMA dispersion becomes worse and overlaps with the increase in the concentration. Consequently, the hydrogen bond formation blocking ability between the stock fibers is weakened, and thus, the sheet tensile strength decreases gradually.

A further comparison reveals that the tensile strength is decreased more by the H-CPGMA replacement than by the S-CPGMA substitution. Our previous study showed that a 0.5% CPGMA substitution significantly improved the dewatering and the wet-end retention performance in CPAM/SiO_2_/APAM papermaking [15]. Figure 3 shows that in the MP(CPGMAs) + GCC + CPAM/SiO_2_/APAM system, a 0.5% S-CPGMA substitution decreases the sheet tensile strength from 32.8 Nm/g to 30.6 Nm/g, indicating a 6.7% decrease, and a 0.5% H-CPGMA substitution decreases the sheet tensile strength from 32.8 Nm/g to 29.8 Nm/g, indicating a decrease of 9.1%. These results showed that when using a small amount of CPGMAs, the sheet tensile strength is weakened in a limited range, while the water filtration performance of the CPAM/SiO_2_/APAM system can be remarkably improved [15].

#### 3.3.2. Bursting Strength

**Figure 4:**
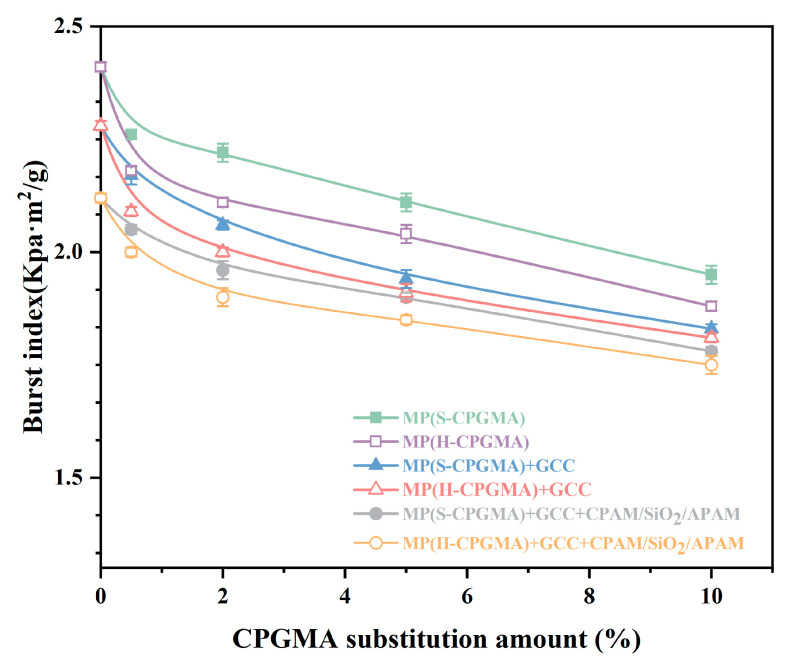
Effect of CPGMA substitution on the sheet bursting strength. (The abbreviations are defined in Table 1 of Section 2.4).

The factors affecting the bursting strength are the bonding force between fibers, fiber length, fiber strength, and fiber arrangement. The impact of the CPGMA replacement quantity on the paper sheet’s bursting strength is shown in Figure 4. When the CPGMA substitution amount is zero, the sheet bursting strength of MP is the highest, followed by MP+GCC. The bursting strength of MP + GCC + CPAM/SiO_2_/APAM is the lowest. The addition of GCC and CPAM/SiO_2_/APAM application decreases the bursting strength mainly because the GCC in the paper reduces the hydrogen bonding between the fibers. The GCC retention in CPAM/SiO_2_/APAM can be further improved, resulting in a decline in the bursting strength. 

In the comparison of the six stock systems, the bursting strength decreases as the substitution amount of S-CPGMAs or H-CPGMAs increases from 0% to 10%. The strength change can be divided into two phases: a rapid case involving the substitution of <0.5% and a slow case involving the substitution of >0.5%. This trend may be attributed to the large change in the dispersion properties of hydrophobic fiber CPGMAs when the substitution amount of CPGMAs is approximately 0.5%, resulting in a significant change in the number of hydrogen bonds formed between the sheet fibers.

From the comparison, it can be further observed that the decrease in strength due to H-CPGMAs was higher than that due to S-CPGMAs. Considering the practical application of CPGMA@CPAM/SiO_2_/APAM, we chose only 0.5% CPGMA substitution and found that the S-CPGMA substitution decreased the bursting index from 2.12 Kpa-m^2^/g to 2.05 Kpa-m^2^/g, indicating a decrease of 3.3%, and the H-CPGMA substitution decreased the bursting index from 2.12 Kpa-m^2^/g to 2.00 Kpa-m^2^/g, indicating a decrease of 5.7%. This indicates that only a small amount of CPGMAs cannot reduce the bursting resistance significantly.

#### 3.3.3. Tearing Resistance

**Figure 5:**
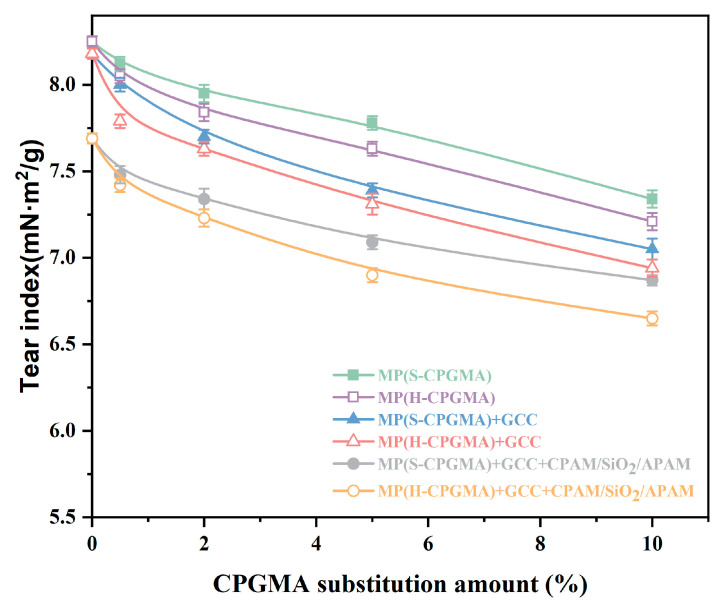
Effect of CPGMA substitution on the sheet tear resistance. (The abbreviations are defined in Table 1 of Section 2.4).

The paper tearing resistance is affected by fiber length, the bonding force between fibers, fiber orientation, fiber strength, and fiber interweaving. The impact of CPGMA replacement on tearing resistance is shown in Figure 5.

In the comparison of the six stock systems, the results showed that the GCC addition and CPAM/SiO_2_/APAM application have different effects on the sheet tearing strength. When the CPGMA substitution amount is zero, the sheet formed by MP is the highest in terms of tearing resistance, the sheet formed by MP + GCC is the second highest, and the sheet formed by MP + GCC + CPAM/SiO_2_/APAM has the lowest tearing resistance. These results are related to the effect of GCC in the paper on the hydrogen bonding between fibers. The GCC retention can be further increased in the CPAM/SiO_2_/APAM, leading to a further decrease in the hydrogen bonding between the sheet fibers, resulting in a further decrease in the tearing resistance.

When the CPGMA substitution increases from 0% to 10%, the tearing resistance decreases as the S-CPGMA or H-CPGMA substitution increases. The tearing resistance change can be divided into two apparent stages: a rapid decrease involving a substitution of <0.5% and a gradual decrease involving a substitution of >0.5%. This trend may be further attributed to the large change in the degree of hydrogen bond formation between sheet fibers when the replacement amount of hydrophobic fiber CPGMAs changes by approximately 0.5%.

In addition, the decrease in the sheet tearing resistance due to H-CPGMAs is higher than that due to S-CPGMAs, which may be related to the length of the fibers. To evaluate the potential of the CPGMA@CPAM/SiO_2_/APAM system for use in practical applications, only 0.5% CPGMA substitution in MP + GCC + CPAM/SiO_2_/APAM was adopted. The S-CPGMAs and H-CPGMAs reduced the tearing strength by 2.7% and 3.5%, respectively. It was inferred that using only a small amount of CPGMAs has little effect on the tearing strength.

#### 3.3.4. Folding Endurance

**Figure 6:**
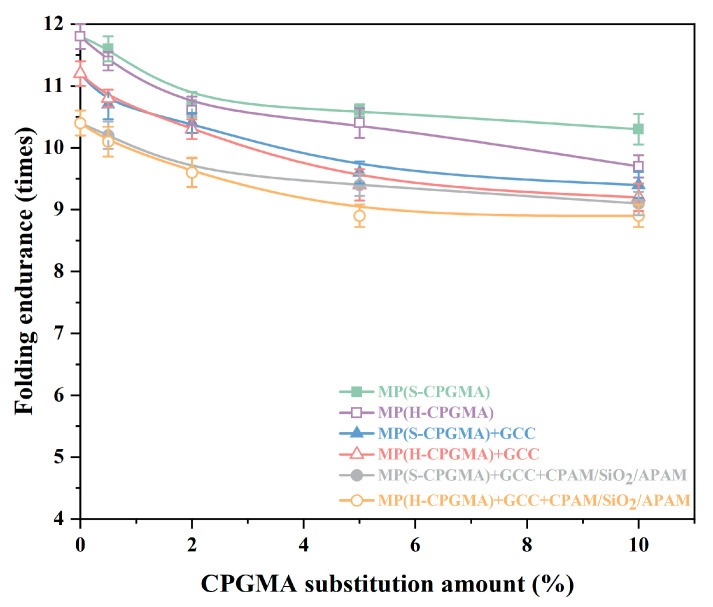
Effect of CPGMA substitution on the sheet folding endurance. (The abbreviations are defined in Table 1 of Section 2.4).

The main factors affecting the sheet folding endurance are fiber length, bonding force between fibers, fiber arrangement, fiber strength, and fiber elasticity. Figure 6 shows the effects of CPGMA substitution on the sheet folding endurance. The sheets formed by MP, MP + GCC, and MP + GCC + CPAM/SiO_2_/APAM without CPGMA substitution demonstrate different folding resistances of 11.8, 11.2, and 10.4 times, respectively. The folding resistance is in the order of MP > MP + GCC > MP + GCC + CPAM/SiO_2_/APAM, which may also be related to the GCC content in the sheet. The higher the GCC content, the weaker the hydrogen bonding between the sheet fibers and the lower the sheet folding endurance.

From the comparison of the six stock systems, it was found that when the CPGMA substitution amount increases from 0% to 10%, the change trend of the sheet folding endurance is similar to that of the sheet tensile strength, bursting strength, and tearing resistance; that is, the folding endurance decreases as the CPGMA substitution increases. Similar to Section 3.3.1, Section 3.3.2 and Section 3.3.3, the change process can also be divided into two stages: a rapid decline involving a substitution amount of <0.5% and a gradual decline involving a substitution amount of >0.5%. Moreover, from the comparison of the six cases, we can find that the H-CPGMAs decrease the folding endurance to a greater extent than S-CPGMAs, which may be related to the fiber length. Considering the practical application of CPGMA@CPAM/SiO_2_/APAM, we used only 0.5% CPGMA substitution in MP + GCC + CPAM/SiO_2_/APAM. In this instance, the changes for S-CPGMAs and H-CPGMAs resulted in a sheet folding endurance that was 10.2 times and 10.1 times, respectively, less than those that did not have such substitutions. This is a drop of 1.92% and 2.88%, respectively. The results show that this did not prove to influence much.

### 3.4. Effects of CPGMA Substitution on Sheet Air Permeability

**Figure 7:**
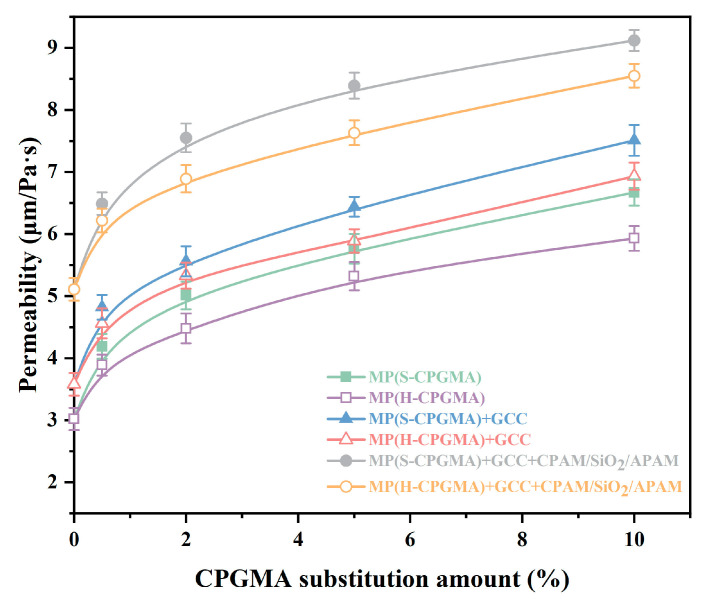
Effect of CPGMA substitution on the sheet air permeability. (The abbreviations are defined in Table 1 of Section 2.4).

Paper must have excellent permeability and stiffness for filter materials, which are often used in special industrial applications. Figure 7 shows the effects of CPGMA substitution on sheet air permeability. The sheets formed by the three stock systems of MP, MP + GCC, and MP + GCC + CPAM/SiO_2_/APAM possess different air permeability. The order of air permeability of MP, MP + GCC, and MP + GCC + CPAM/SiO_2_/APAM is MP + GCC + CPAM/SiO_2_/APAM > MP + GCC > MP, which is 3.02 μm/Pa·s, 3.58 μm/Pa·s, and 5.11 μm/Pa·s, respectively. The air permeability of a paper sheet is related to the space. The space is related to the bonding strength and the number of bonds between the fibers. The larger the bonding strength and the higher the number of sites, the denser the paper and the smaller the air permeability. GCC in the sheet reduces the bonding strength and the site number between fibers. The reduction in bonding strength and site number between fibers increases with increasing GCC concentration. This further increases the gap and improves the air permeability. Therefore, the GCC retention and the sheet air permeability of the CPAM/SiO_2_/APAM system are observed to be the highest. The sheet air permeability of CPAM/SiO_2_/APAM is 69% higher than that of the sheet of MP.

Figure 7, which compares the six stock systems, shows that as the CPGMA substitution amount grows from 0% to 10%, so does the air permeability of the sheet generated by these systems. The increasing trend can be divided into a rapid stage involving a substitution amount of <0.5% and a gradual stage involving a substitution amount of >0.5%. The results may be related to the strong hydrophobicity [13] and high stiffness [14] of CPGMAs. In addition, from the comparison of the six cases, the decrease in the sheet air permeability due to S-CPGMAs is observed to be higher than that due to H-CPGMAs mainly because S-CPGMAs are longer than H-CPGMAs [13,14]. Considering the practical application of CPGMA@CPAM/SiO_2_/APAM technology, only 0.5% CPGMA was added, and we found that 0.5% S-CPGMA increased the sheet air permeability from 5.11 μm/Pa·s to 6.49 μm/Pa·s, indicating an increase of 27.0%. The H-CPGMAs increased the sheet air permeability from 5.11 μm/Pa·s to 6.22 μm/Pa·s, indicating an increase of 21.7%. The results show that a small amount of CPGMAs can improve the paper air permeability, which is essential for some specialty papers.

## 4. Conclusions

The distinct improvement in the hydrophobicity of fibers is favorable for the water filtration performance but weakens the bonding performance between fibers. We studied the effects of the amount of pulp substitution by CPGMAs in the stocks on the sheet properties in the CPAM/SiO_2_/APAM system and obtained the following conclusions:

(1) As the percentage of CPGMA replacement in the stock rose, the sheet’s tensile strength, bursting strength, tearing strength, and folding endurance all reduced.
(2) The paper air permeability increased with the CPGMA substitution amount in the stock. A small amount of CPGMAs can distinctly improve the paper’s air permeability.
(3) The substitution of fibers with CPGMAs in the stock had little effect on the paper formation.

A small amount of CPGMAs greatly enhances certain paper characteristics and the efficiency of the paper production process, but it may have a limited effect on the paper’s strength. The CPGMAs@CPAM/SiO_2_/APAM system could be a potential industrial application, further improving the filtration and retention of the CPAM/SiO_2_/APAM system. Subsequent research in this area can be expanded to include the effect of pulp fiber replacement on the properties of various papers. At the same time, in-depth studies of the effects of different substitution rates and different CPGMA types on paper properties can be planned, allowing staff to fine-tune and improve practical applications. Ultimately, the overall requirements of strengthening retention and filtration properties, improving paper performance, promoting environmentally friendly papermaking, reducing water consumption and production costs, and reducing environmental pollution will be achieved.

## Figures and Tables

**Table 1:** Handsheet stock compositions.

	SBKP (%)	HBKP (%)	BCTMP (%)	GCC (%) (/g MP)	CPAM/SiO_2_/APAM (ppm)	S-CPGMA (%)	H-CPGMA (%)
MP	28.0	52.0	20.0	-	-	-	-
MP (S-CPGMA)	27.5/26.0/23.0/18.0	52.0	20.0	-	-	0.5/2.0/5.0/10.0	-
MP (H-CPGMA)	28.0	51.5/50.0/47.0/42.0	20.0	-	-	-	0.5/2.0/5.0/10.0
MP + GCC	28.0	52.0	20.0	20.0	-	-	-
MP + GCC (S-CPGMA)	27.5/26.0/23.0/18.0	52.0	20.0	20.0	-	0.5/2.0/5.0/10.0	-
MP + GCC (H-CPGMA)	28.0	51.5/50.0/47.0/42.0	20.0	20.0		-	0.5/2.0/5.0/10.0
MP + GCC + CPAM/SiO_2_/APAM	28.0	52.0	20.0	20.0	300/3600/250	-	-
MP + GCC + CPAM/SiO_2_/APAM (S-CPGMA)	17.5/26.0/23.0/18.0	52.0	20.0	20.0	300/3600/250	0.5/2.0/5.0/10.0	-
MP + GCC + CPAM/SiO_2_/APAM (H-CPGMA)	28.0	51.5/50.0/47.0/42.0	20.0	20.0	300/3600/250	-	0.5/2.0/5.0/10.0

The abbreviations are as follows: SBKP, softwood bleached kraft pulp; HBKP, hardwood bleached kraft pulp; BCTMP, bleached chemical–thermal–mechanical pulp; GCC, ground calcium carbonate; CPAM/SiO_2_/APAM, cationic polyacrylamide/colloidal SiO_2_/anionic polyacrylamide; S-CPGMA, polyglycidyl methacrylate-grafted softwood kraft pulp fiber; H-CPGMA, polyglycidyl methacrylate-grafted hardwood kraft pulp fiber; MP, mixed pulp; MP(S-CPGMA), mixed pulp (polyglycidyl methacrylate-grafted softwood kraft pulp fiber); MP(H-CPGMA), mixed pulp (polyglycidyl methacrylate-grafted hardwood kraft pulp fiber); MP + GCC, mixed pulp with only ground calcium carbonate; MP + GCC(S-CPGMA), mixed pulp with only ground calcium carbonate (polyglycidyl methacrylate-grafted softwood kraft pulp fiber); MP + GCC(H-CPGMA), mixed pulp with only ground calcium carbonate (polyglycidyl methacrylate-grafted hardwood kraft pulp fiber); MP + GCC + CPAM/SiO_2_/APAM, mixed pulp with ground calcium carbonate and cationic polyacrylamide/colloidal SiO_2_/anionic polyacrylamide system; MP+GCC+CPAM/SiO_2_/APAM(S-CPGMA), mixed pulp with ground calcium carbonate and cationic polyacrylamide/colloidal SiO_2_/anionic polyacrylamide system (polyglycidyl methacrylate-grafted softwood kraft pulp fiber); MP + GCC + CPAM/SiO_2_/APAM(H-CPGMA), mixed pulp with ground calcium carbonate and cationic polyacrylamide/colloidal SiO_2_/anionic polyacrylamide system (polyglycidyl methacrylate-grafted hardwood kraft pulp fiber).

## Data Availability

The article contains data.
